# Proxying credit curves via Wasserstein distances

**DOI:** 10.1007/s10479-022-04552-3

**Published:** 2022-02-16

**Authors:** Matteo Michielon, Asma Khedher, Peter Spreij

**Affiliations:** 1 Quantitative Analysis and Quantitative Development, ABN AMRO Bank N.V., Gustav Mahlerlaan 10, 1082 PP Amsterdam, The Netherlands; 2 Korteweg-de Vries Institute for Mathematics, University of Amsterdam, Science Park 105-107, 1098 XG Amsterdam, The Netherlands; 3 Institute for Mathematics, Astrophysics and Particle Physics, Radboud University Nijmegen, Huygens Building, Heyendaalseweg 135, 6525 AJ Nijmegen, The Netherlands

**Keywords:** Credit default swap, Harmonic mean, Hazard rate, Proxy credit curve, Wasserstein barycenter, Wasserstein distance

## Abstract

Credit risk plays a key role in financial modeling, and financial institutions are required to incorporate it in their pricing, as well as in capital requirement calculations. A common manner to extract credit worthiness information for existing and potential counterparties is based on the Credit Default Swap (CDS) market. Nonetheless, not all counterparties of a financial institution have (liquid) CDSs traded in the market. In this case, financial institutions shall employ a proxy methodology to estimate the default probabilities of these counterparties. Starting from the intersection methodology for credit curves, in this article we investigate whether it is possible to construct proxy credit curves from CDS quotes by means of (weighted) Wasserstein barycenters. We show how, under simple and common assumptions, this revised methodology leads to elementary and intuitive formulae to calculate distances between CDS-implied default probability distributions. Further, we illustrate how to use this information to construct proxy CDS quotes.

## Introduction

In this article we investigate an alternative approach to construct proxy credit curves starting from Credit Default Swaps (CDSs) market data. In particular, we revise the *intersection methodology* by calculating the (weighted) *Wasserstein barycenter* of the implied CDS credit curves considered as inputs. Under simple assumptions our new methodology results in analytical formulae for the proxy CDS hazard rates with an intuitive interpretation.

Banks are required, for instance in valuation adjustment, risk limits and capital calculations, to account for credit risk as accurately as possible. Nonetheless, not for all counterparties credit-related information can be (easily) extracted from financial instruments traded in the market. For instance, simple instruments used to strip default probabilities of given counterparties are CDSs. However, not all counterparties have liquid CDSs trading in the market, and many have no CDSs trading at all. Therefore, proxy methodologies should be employed, as for instance the intersection methodology described in EBA (2013) (see also Chourdakis et al. (2013a) and Sourabh et al. (2018) for further details). In this approach the proxy spread for an illiquid entity with a given rating, region, sector, etc. is calculated as the average of all the available liquid spreads of the entities with the same characteristics, i.e., rating, region, sector, etc.^1^ In this article we investigate whether the Wasserstein square distance can be considered as a tool to substitute the aforementioned average in the intersection model. In particular, we show how using the Wasserstein distance allows, given some simple assumptions, to obtain proxy CDS curves in a simple way.

The modeling of the default time of a counterparty within the risk-neutral framework can be easily embedded in pricing and hedging equations; see Jarrow and Turnbull (1995) and Ammann (1999, Sect. 5), amongst others. However, this is based on the implicit assumption that faithful credit data is available. Unfortunately, this is not necessarily guaranteed in practice. In fact, see Green (2016, Sect. 4.6), the number of counterparties worldwide for which CDS quotes are available is in the order of a few thousand and, further, not all the available quotes are liquid enough to be considered trustworthy. Therefore, it does not come as a surprise that, for the most of the counterparties of financial institutions, no (liquid) CDSs are available to imply the required default probabilities. From this, the need of using a CDS proxy methodology to compensate for the lack of available (or reliable) data arises.

Despite the fact that financial institution can decide, provided approval is granted by validators, auditors and regulators, for the methodology to be used to proxy CDS curves, the literature is quite scarce. For instance, financial institutions can map the missing or unreliable CDS data needed for given counterparties to that of other counterparties for which liquid single-name CDS data is available, based on selection criteria such as rating, region, sector, etc.; see Green (2016, Sect. 4.6.1). This has the advantage that, if the mapping selection is done carefully, then the proxy curves would reflect the same rating-, region- and sector-specific risks of the company whose CDS curve needs to be proxied. From a theoretical perspective, this would allow to hedge the credit risk by taking positions in the mapped CDS. However, this would not guarantee synchronicity of default or credit downgrades/upgrades (if any) between the illiquid counterparty and its proxy. This might result in unexpected gains or losses, as well as in potential re-hedging costs. Alternatively, see Green (2016, Sect. 4.6.2), missing single-name CDS data can be mapped to given CDS indices. Notwithstanding, this can lead to similar situations as the one highlighted in the single-name mapping case. A way to construct proxy CDS curves that does not involve simply mapping missing CDS data to that of other companies or indices is the so-called intersection methodology (EBA 2013). This approach suggests to bucket liquid CDSs according to their rating, region and sector. By averaging the CDS quotes in each bucket, one can then define proxy CDS quotes per bucket and maturity (see also Chourdakis et al. (2013a) and Sourabh et al. (2018)). This approach is very intuitive and easy to implement. Also, should one need to make the approach more granular, then this could be easily achieved by considering a finer bucketing methodology.^2^ However, the intersection methodology has the disadvantage that, in some cases, not many CDS names are available with a certain rating, region and sector, which might cause some buckets to only contain a handful of quotes (or even to be empty). We also recall the so-called *cross-sectional methodology* developed in Chourdakis et al. (2013a) (see also Chourdakis et al. 2013b and Green 2016, Sect. 4.6.3). This methodology assumes that each CDS spread can be decomposed in a number of factors, which are then calibrated to the available CDS spreads by means of least-square regression. This approach has the advantage, as its name suggests, of taking into account the cross-sectional information of CDS spreads arising from different ratings, regions and sectors. The cross-sectional methodology of Chourdakis et al. (2013a) has been extended in Sourabh et al. (2018) in order to potentially include equity returns in the regression to further enhance the reliability and stability of the results.

In this article we consider the intersection methodology as a starting point, and we look at it from another angle. Assuming the available CDS quotes have been allocated according to a given bucketing criterion we investigate whether, instead of averaging the quotes, it is possible to create CDS curves in a different manner. In particular, we attempt to do so by means of calculating Wasserstein square distances between the CDS-implied default probability curves corresponding to the instruments allocated to the same bucket. We show that, under some well-known approximations, this is possible. This leads to analytic formulae for the hazard rates of the proxy CDSs, which are also intuitive from the interpretation angle.

This article is organized as follows. Section 2 provides the essential concepts on Wasserstein distances relevant for the methodology developed in this article, and it also outlines the basic notions related to CDSs. In Sect. 3 the methodology to proxy CDS curves using Wasserstein square distances is available. In Sect. 4 we comment on the possible use of some alternative metrics between probability distributions for the purpose of proxying CDS curves and further motivate our choice. In Sect. 5 two extensive examples of proxy CDS quotes construction are provided. Section 6 concludes.

## Background information

In this section we outline the basic notions which are needed throughout this article. In particular, Sect. 2.1 provides the essential background concerning Wasserstein distances, while Sect. 2.2 that on CDSs. In both Sects. 2.1 and 2.2 the interested reader will find relevant references should more detailed and general information be needed.

### A brief overview of Wasserstein distances

In this section, we provide the essential notions concerning Wasserstein distances as necessary in the rest of the article. For an in-depth treatment of Wasserstein distances see Villani (2003, Sect. 7, 2009, Sect. 6), or Panaretos and Zemel (2020, Sect. 1 and 2), where Wasserstein distances are treated from a more general angle covering aspects that go beyond our needs in this article.

The concept of Wasserstein distance finds its origin in the area of optimal transport. In particular, it aims to measure the minimal work that is required to rearrange the probability masses of one distribution in order to reconstruct a second distribution. This transportation plan should be performed in order to minimize the cumulative “distance” that the various probability masses have to go through.

Given any two probability measures on $\mathbb {R}^d$ denoted with $\mathbb {P}$ and $\mathbb {Q}$, we indicate with $\mathcal {J}(\mathbb {P}, \mathbb {Q})$ the set containing all the joint distributions which have $\mathbb {P}$ and $\mathbb {Q}$ as marginals. For $p\ge 1$, the *p*-Wasserstein distance between $\mathbb {P}$ and $\mathbb {Q}$ is defined as

1 $$\begin{aligned} W_p(\mathbb {P}, \mathbb {Q}){:}{=}\left( \inf _{J\in \mathcal {J}(\mathbb {P}, \mathbb {Q})} \int _{\mathbb {R}^d\times \mathbb {R}^d}\Vert x-y\Vert ^{p}\,J(dx\times dy)\right) ^{\frac{1}{p}}, \end{aligned}$$

where $\Vert \cdot \Vert$ denotes the Euclidean norm. The Wasserstein distance (1) is finite when the distributions have finite moments of order *p*; see Panaretos and Zemel (2019). When $p=2$, (1) is referred to as *Wasserstein square distance*, which is the analogous in a probabilistic sense of the Euclidean space distance. In the case of unidimensional random variables (1) can be easily calculated via the relationship

2 $$\begin{aligned} W_p(\mathbb {P}, \mathbb {Q})=\left( \int _0^1|F_{\mathbb {P}}^{-1}(u)-F_{\mathbb {Q}}^{-1}(u)|^p\,du\right) ^{\frac{1}{p}}, \end{aligned}$$

where $F_{\mathbb {P}}^{-1}(\,\cdot \,)$ ($F_{\mathbb {Q}}^{-1}(\,\cdot \,)$) denotes the (pseudo-)inverse of the cumulative distribution function of $\mathbb {P}$ ($\mathbb {Q}$); see Panaretos and Zemel (2019). In the present article, we will be interested in the unidimensional case only and, in particular, in the case when $p=2$.

We recall now the concept of *weighted Wasserstein barycenter*, as it will be useful for an analogy we will provide in Sect. 3. Given *N* probability measures $\mathbb {P}_1,\ldots ,\mathbb {P}_N$ with the characteristics as above and positive weights $w_1,\ldots ,w_N$ such that $\sum _{i=1}^Nw_i = 1$, a Wasserstein barycenter, see Agueh and Carlier (2011), is any probability distribution $\mathbb {P}^*$ solution of

3 $$\begin{aligned} \inf \left\{ \sum _{i=1}^N w_iW_2^2(\mathbb {P}_i, \mathbb {P}) : \mathbb {P} \text { has a finite second moment}\right\} . \end{aligned}$$

For practical applications, we recall that if the probabilities $\mathbb {P}_i$’s are absolutely continuous, then both existence and uniqueness of the Wasserstein barycenter are guaranteed; see Agueh and Carlier (2011).^3^ Often, the definition of weighted Wasserstein barycenter is provided with uniform weights, i.e., with $w_i{:}{=}\frac{1}{N}$ for every *i*. In that case, the dependence on $w_1,\ldots ,w_N$ can be then simply omitted.^4^

The popularity of Wasserstein distances has increased considerably in the last decade, as well as the number of applications of Wasserstein distances in different fields. This is due to some desirable properties Wasserstein distances exhibit, including, but not limited to, their behavior along geodesics (see Ambrosio et al. 2008, Sect. 7), and to Wasserstein distances incorporating the geometry of the ground space (see Panaretos and Zemel 2019). We also recall the use of Wasserstein distances in statistical learning (Karimi et al. 2020), auto-encoders (Zhang et al. 2021), martingale optimal transport (Guo and Obłój 2019), and image labelling (Hühnerbein et al. 2018), to name but a few. For computational challenges concerning Wasserstein barycenters see Altschuler and Boix-Adserà (2021a; b), while for an overview of optimal transport theory from a computational angle with applications in machine learning see Peyré and Cuturi (2019).

### A brief overiew of CDSs

A CDS is a credit derivative contract involving two counterparties: a protection buyer and a protection seller. In particular, by entering the transaction the protection buyer obtains protection against a contract-specific credit event (e.g., a credit rating downgrade or a default) concerning a given reference entity. In exchange for holding the credit risk, the protection seller expects the counterparty to pay a recurrent protection fee until either the contract expires or a credit event occurs. CDSs are nowadays traded given an upfront premium and a fixed coupon. The CDS market provides an important source of data that can be used to estimate the default probabilities of different entities. These default probabilities can be then employed in various areas related to credit risk modeling, including valuation adjustment calculations.

The process of extracting default probabilities from CDS quotes can be performed in different manners. A simple and effective way to do so is by means of *reduced-form* models, which are based on the idea of assigning a given functional form for the default probability function and to calibrate its parameters in order to reprice the available CDS contracts in the market (for a detailed overview of several approaches concerning modelling default probabilities see Bielecki and Rutkowski 2004). In particular, a simple manner to model the default probability of an entity is that of assuming its (risk-neutral) default probability function to be defined via the relationship

4 $$\begin{aligned} \mathbb {Q}\left( \tau \le t\right) {:}{=}1- e^{-\int _0^t \lambda (s)\,ds}. \end{aligned}$$

In (4), $\mathbb {Q}$ denotes the risk-neutral measure, $\tau$ the (random) default time of the entity, and the (deterministic) function $\lambda :(0,+\infty )\rightarrow (0,+\infty )$ is named *hazard rate* (or *default intensity*).^5^ Different specifications for the hazard rate function can be chosen: common ones are flat, piecewise-constant, piecewise-linear and cubic spline.

We now recall some simple and well-known approximations for hazard rates, known as *credit triangles*, which are often used in practice (see, amongst others, Berd 2005, 2011; White 2014; Gambetti et al. 2018 and Spiegeleer et al. 2014, Sect. 10.4 for further details). The CDS *par spread* is defined as the coupon of the fixed leg that would make the CDS contract trade at par. This used to be the standard way of quoting CDSs. Having denoted the recovery rate of a given CDS as *R*^6^ and its par spread as *s*, assuming the hazard rate function in Eq. (4) is flat, one can then approximate the constant hazard rate in (4) via^7^

5 $$\begin{aligned} \lambda \approx \frac{s}{1-R}. \end{aligned}$$

This means that the distribution of the default time $\tau$ is modeled by means of an *exponential distribution* with *rate parameter* $\lambda$. Note that, as a consequence of the CDS standardization process that took place after the credit crisis (see, amongst others, Markit 2009a, b), nowadays it is market practice to quote CDSs is by means of a standard coupon and an upfront payment. In this case, the credit triangle relationship would read $\lambda \approx (c + \mathrm {PUF}/T)/(1-R)$, where *c* denotes the standard coupon of the CDS, $\mathrm {PUF}$ the *points up-front*, i.e., the upfront payment quoted as percentage of the notional, while *T* the time to maturity of the contract considered.^8^ The simplifying assumption of a constant hazard rate is often used in practice to convert from one quoting convention (i.e., points upfront and standard coupon) to the other (i.e., par spread); see White (2014).

## CDS proxy curves using Wasserstein square distances

Assume we have grouped liquid CDS quotes given some bucketing criteria (e.g., their sector, region, etc.), and that one bucket for which we want to extract a proxy CDS curve has been selected. For each of the CDSs in the group we have a set of maturities. We choose a maturity amongst the ones available, as the process can be performed for each maturity separately. We assume that $N\ge 2$ par spreads are available (see Sect. 2.2), and we denote them as $s_1,\ldots ,s_N$ (we could follow a similar approach if we decided to start from upfront premia instead of from par spreads; again, see Sect. 2.2). Thus, for each of the CDS quotes in this group (recall the maturity is fixed) we have a par spread. For each CDS we now compute the *flat* hazard rate via the credit triangle relationship (5). The approximation of having a flat hazard rate per CDS basically says that, once a CDS is considered, this CDS belongs to an economy composed by the risk-free bond and the CDS itself. Thus, the easiest way to calculate a hazard rate is by assuming it flat, as done in Sect. 2.2. This assumption is a simplifying one, as it does not take into account the term structure of the hazard rates for different CDS maturities. However, it is a common approximation used in practice, as for instance credit triangles are based on this assumption; see (5). For our group of *N* CDSs, we use the assumption of a constant hazard rate, and thus for each par spread $s_i$ we can calculate a flat hazard rate $\lambda _i$. This approximation based on a constant hazard rate assumption is the same used to perform the conversion between upfront payments and related par spreads; see White (2014). This approximation is also used, for instance, in Madan (2014) in the context of measuring risk acceptability within the CDS market. The reason why we propose to use this simplifying assumption is that it allows to exactly calculate proxy hazard rates. More precisely, this is because having a constant hazard rate per maturity allows to analytically calculate Wasserstein distances between CDS-implied distributions, as shown in Theorem 1. The distribution function of the default time implied from the *i*th CDS is denoted as $F_{\mathbb {Q}_i}(t){:}{=}\mathbb {Q}(\tau _i\le t)$, with $\tau _i$ indicating the default time of the *i*th reference entity. For the *i*th CDS, we can calculate the inverse of the default probability distribution explicitly, i.e., $F_{\mathbb {Q}_i}^{-1}(u) =-\frac{\ln (1-u)}{\lambda _i}$ by inverting (4). We can consider an additional CDS (i.e., the *proxy* CDS) which, under the same assumptions of constant hazard rate, defines the quantile function $F_{\mathbb {Q}^*}^{-1}(u) =-\frac{\ln (1-u)}{\lambda }$ for a given constant $\lambda$ to be determined. We can then calculate the *weighted square error* in Wasserstein square distance between the distribution of the proxy CDS $\mathbb {Q}^*$ and those of the other *N* CDSs $\mathbb {Q}_1,\ldots ,\mathbb {Q}_N$, i.e.,

6 $$\begin{aligned} wse(\lambda ){:}{=}\sum _{i=1}^N w_i W_2^2(\mathbb {Q}_i, \mathbb {Q}^*), \end{aligned}$$

where $\sum _{i=1}^N w_i=1$ and with $w_i>0$ for every *i*.^9^ By minimizing (6) with respect to $\lambda$ we can then obtain the optimal hazard rate. Note the similarity of (6) with the definition of Wasserstein barycenter (3): it is clear how (6) represents the Wasserstein barycenter between the probabilities $\mathbb {Q}_i$’s after a functional form for the barycenter itself has been chosen. Therefore, the probability measure $\mathbb {Q}^*$ in (6) can be interpreted as a *pseudo* Wasserstein barycenter for the default distributions we have considered.

We now provide an analytical expression for the optimal hazard rate in Theorem 1, which illustrates how the optimal hazard rate is actually the *weighted harmonic mean* of the hazard rates $\lambda _i$’s. Note that, from here onward, the index *i* will be always assumed to range in $\left\{ 1,\ldots ,N\right\}$, for ease of exposition.

### Theorem 1

Let $\mathbb {Q}_1,\ldots , \mathbb {Q}_N$ be exponential distributions where, for every *i*, $\mathbb {Q}_i$ has rate parameter $\lambda _i$. Further, let $\mathbb {Q}^*$ be another exponential distribution with rate parameter

7 $$\begin{aligned} {\lambda ^{*}} {:}{=}\frac{1}{\sum _i \frac{w_i}{\lambda _i}}. \end{aligned}$$

Then, $\lambda ^*$ is the unique minimizer of $wse(\,\cdot \,)$.

### Proof

Recalling that the quantile function of an exponential distribution $\mathbb {Q}$ with rate parameter $\lambda$ is given by $F_{\mathbb {Q}}^{-1}(u) =-\frac{\ln (1-u)}{\lambda }$, it results that

8 $$\begin{aligned} W^2_2(\mathbb {Q}_i, \mathbb {Q})=\int _0^1\left( \frac{\ln (1-u)}{\lambda _i} -\frac{\ln (1-u)}{\lambda } \right) ^2\,du=2\left( \frac{1}{\lambda _i} -\frac{1}{\lambda } \right) ^2. \end{aligned}$$

Let $x{:}{=}\frac{1}{\lambda }$. It is sufficient to consider the function

$$\begin{aligned} f(x) {:}{=}\sum _i w_i\left( \frac{1}{\lambda _i} -x \right) ^2 \end{aligned}$$

and to observe that $\frac{d}{dx}f(x)=0$ when $x=\frac{1}{\lambda ^*}$, while $\frac{d^2}{dx^2}f(x)\equiv 2$ due to the weights $w_i$’s summing up to one. $\square$

Note that the mean of $\mathbb {Q}_i$ equals $\frac{1}{\lambda _i}$. Thus, due to Theorem 1 the reciprocal of the proxy hazard, i.e., $\frac{1}{\lambda ^*}$ can be interpreted as the weighted average expectation of the *N* default probability distributions considered. This means that the weighted harmonic mean of the $\lambda _i$’s is actually the correct way of averaging them, as they can be interpreted as rates.

We highlight that using flat hazard rates is the key simplifying hypothesis that guarantees the simple formula of the optimal hazard rate (7) to hold. If this assumption were to be dropped and a more elaborate functional form for the hazard rate term structure were to be chosen, then a closed form for computing the optimal (weighted sum of) Wasserstein distance(s) (6) would not be available. Instead, it would require to solve a minimization problem in several unknowns (i.e., one per CDS maturity available), making the whole procedure more complicated and computationally expensive. More precisely, given *N* CDS names available per maturity, let *M* denote the total number of quoted CDS maturities (*M*, in many case would be equal to 11, as ideally CDS quotes are available for the 6M, 1Y, 2Y, 3Y, 4Y, 5Y, 7Y, 10Y, 15Y, 20Y and 30Y maturities). Assume that default probabilities were to be modeled by means of (4) in a more elaborate manner such as piecewise-constant or piecewise-linear.^10^ Denote with $\lambda _i^j$ the hazard rate corresponding to the *i*th CDS name and to the *j*th maturity. Also, denote with $F^{-1}_{\mathbb {Q}_i}(\lambda _1^i,\ldots ,\lambda _M^i;\,\cdot \,)$ the quantile function implied from the *i*th CDS, and with $F^{-1}_{\mathbb {Q}^*}(\lambda _1,\ldots ,\lambda _M;\,\cdot \,)$ the quantile function generated by the (unknown) proxy CDS yet to be estimated. One would then need to find a solution, via (2), of the constrained optimization problem

9 $$\begin{aligned} \min _{\lambda _1,\ldots ,\lambda _M} \sum _i \int _0^1\left( F^{-1}_{\mathbb {Q}_i}(\lambda _1^i,\ldots ,\lambda _M^i;u)-F^{-1}_{\mathbb {Q}^*}(\lambda _1,\ldots ,\lambda _M;u)\right) ^2\,du, \end{aligned}$$

where each $\lambda _i$ is required to be positive. First of all, it is not guaranteed that, for arbitrary choices of the hazard rate function, a unique solution exists. Furthermore, the integrand in (9) cannot be, in general, integrated analytically, and the relatively high number of degrees of freedom (i.e., *M*), together with the potentially high number of CDSs in the bucket considered, would make the problem very expensive from the computational angle. Thus, these observations tip the balance in favor of a simpler approach, as the one we have adopted involving a constant hazard rate.

We now provide a second intuitive result highlighting some relationship between the observed hazard rates (implied default distributions) and its optimal counterpart. Its proof is trivial, and it directly follows from the interpretation of the optimal hazard rate $\lambda ^*$ as an harmonic mean and from the (strict) monotonicity of the distribution function of an exponential random variable with respect to the rate parameter. For ease of readability, we denote with $\lambda _{\mathrm {min}}{:}{=}\min _i\lambda _i$ and with $\lambda _{\mathrm {max}}{:}{=}\max _i\lambda _i$, while with $i_{\mathrm {min}}$ and $i_{\mathrm {max}}$ the corresponding indices, i.e., such that $\lambda _{i_\mathrm {min}}=\lambda _{\mathrm {min}}$ and $\lambda _{i_\mathrm {max}}=\lambda _{\mathrm {max}}$.

### Corollary 1

Under the assumptions of Theorem 1 it holds that

10 $$\begin{aligned} \lambda _{\mathrm {min}} \le \lambda ^*\le \lambda _{\mathrm {max}}. \end{aligned}$$

Furthermore, for every $t\in [0,+\infty )$ it results that

11 $$\begin{aligned} F_{\mathbb {Q}_{{i_{\mathrm {min}}}}} (t)\le F_{\mathbb {Q}^*}(t)\le F_{\mathbb {Q}_{{i_{\mathrm {max}}}}}(t). \end{aligned}$$

So far, we have defined the probability distribution $\mathbb {Q}^*$ as the pseudo-barycenter generated by considering $\mathbb {Q}_1,\ldots ,\mathbb {Q}_N$. We now show that $\mathbb {Q}^*$ represents the actual Wasserstein barycenter of the probability distributions considered.

### Theorem 2

Under the assumptions of Theorem 1 it holds that

12 $$\begin{aligned} \sum _i w_i W_2^2(\mathbb {Q}_i, \mathbb {Q}^*)=\min \left\{ \sum _i w_i W_2^2(\mathbb {Q}_i, \mathbb {Q}) : \mathbb {Q} \text { has a finite second moment}\right\} . \end{aligned}$$

### Proof

We denote with $F_{\mathbb {Q}}^{-1}(\,\cdot \,)$ the quantile function of a given probability distribution $\mathbb {Q}$ with finite second moment, and define

13 $$\begin{aligned} \varDelta _\mathbb {Q}{:}{=}\sum _i w_i W_2^2(\mathbb {Q}_i, \mathbb {Q})=\sum _i w_i\int _0^1\left( F_{\mathbb {Q}_i}^{-1}(u) -F_{\mathbb {Q}}^{-1}(u)\right) ^2\,du. \end{aligned}$$

By adding and subtracting $F_{\mathbb {Q}^*}^{-1}(\,\cdot \,)$ inside the brackets in the third member of (13) it results that

$$\begin{aligned} \varDelta _\mathbb {Q}&= \sum _i w_i \int _0^1\left( F_{\mathbb {Q}_i}^{-1}(u)-F_{\mathbb {Q}^*}^{-1}(u)\right) ^2\,du + \int _0^1\left( F_{\mathbb {Q}^*}^{-1}(u)-F_{\mathbb {Q}}^{-1}(u)\right) ^2\,du \\&\qquad + 2\sum _iw_i \int _0^1\left( F_{\mathbb {Q}_i}^{-1}(u)-F_{\mathbb {Q}^*}^{-1}(u)\right) \left( F_{\mathbb {Q}^*}^{-1}(u)-F_{\mathbb {Q}}^{-1}(u)\right) \,du. \end{aligned}$$

Observe that, by definition of the optimal hazard rate $\lambda ^*$, the quantity $\sum _i w_i(F_{\mathbb {Q}_i}^{-1}(u)-F_{\mathbb {Q}^*}^{-1}(u))$ equals zero. From this it follows that

$$\begin{aligned} \varDelta _\mathbb {Q} = \sum _i w_i W_2^2(\mathbb {Q}_i, \mathbb {Q}^*)+ \int _0^1\left( F_{\mathbb {Q^*}}^{-1}(u)-F_{\mathbb {Q}}^{-1}(u)\right) ^2\,du\ge \sum _i w_i W_2^2(\mathbb {Q}_i, \mathbb {Q}^*). \end{aligned}$$

From the arbitrariness of $\mathbb {Q}$ and from Theorem 1, equality (12) follows. $\square$

For a given group of CDSs one can calculate, for each maturity, the optimal hazard rate $\lambda ^*$ minimizing the squared error in the Wasserstein square distance (6). The corresponding “optimal” par credit spreads, per maturity, can then be implied. This can be done, for instance, by again employing the credit triangle relationship (5), and by assuming a recovery rate equal either to a prespecified value or calculated as the average (e.g., arithmetic or harmonic) of the recovery rates of the CDSs belonging to the same bucket.

Assume now, for simplicity, that for all the CDSs in a given bucket the recovery rate is constant.^11^ If we define the proxy recovery rate as the average (arithmetic or harmonic) of the observed recovery rates and compute the proxy par spread by inverting (5), it then still follows that this CDS par spread equals the harmonic mean of the CDS spreads of the single contracts in the bucket. We recall that the harmonic mean of a set of (non-zero) numbers is at most equal to its arithmetic counterpart due to Jensen’s inequality. While the arithmetic mean equally averages all the data points, which makes it give less importance to observations with very small magnitude, by considering the harmonic mean higher weights are given to those observations. Hence, it then follows that, under the constant recovery rate assumption, the methodology proposed here is less conservative than simply averaging the observed CDS quotes, as well as less (more) sensitive to large (small) outliers.

From a computational perspective, the methodology highlighted in the present article is simple. As Theorem 1 shows, calculating the optimal hazard rates only requires the computation of a weighted harmonic mean. This also allows to interpret the proxy CDS curves in terms of Wasserstein barycenters in virtue of Theorem 2. However, we provide the following remark concerning the interpretation of the technique we have used in this article. Calculating the proxy spread by simply averaging the relevant CDS quotes is an elementary methodology, which very simple to apply and very intuitive. However, calculating the average of the observed spreads is a procedure that is only based on the magnitude of the observations and that does not take into account how the implicit default distributions implied from the different observations interact with each other. On the other hand, the approach we have proposed here bases the construction of the proxy quotes on the implicit distributions arising from the different quotes. This is done by means of finding a new distribution that is as close as possible to the observed ones from an optimal transportation perspective. This gives a fresh view to credit curve proxy methodologies by means of attempting to tackle the problem not from a data perspective but, instead, by involving metrics between probability distributions.

## Suitability of other metrics between probability distributions to proxy CDS curves

**Fig. 1:**
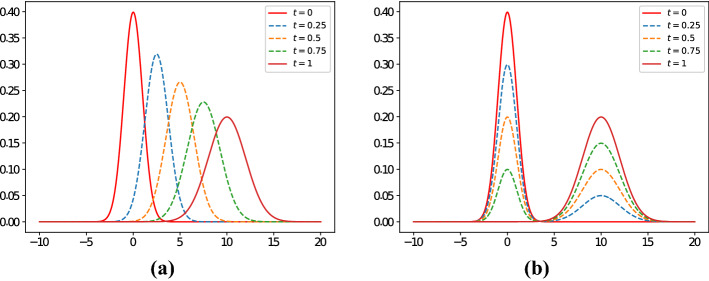
Example of geodesics between probability density functions. In particular, panel **a** represents the geodesic in Wasserstein square distance between a normal density function with mean parameter $\mu _0=0$ and standard deviation $\sigma _0=1$ (denoted with $n_0(\,\cdot \,)$) and a normal density function with mean parameter $\mu _1=10$ and standard deviation $\sigma _1=2$ (denoted with $n_1(\,\cdot \,)$). For each $t\in [0,1]$ the geodesic can be explicitly calculated (see Malagó et al. 2018), and its density (denoted with $n_t(\,\cdot \,)$) corresponds to that of a normal distribution with mean $\mu _t{:}{=}(1-t)\mu _0+t\mu _1$ and standard deviation $\sigma _t{:}{=}(1-t)\sigma _0 + t\sigma _1$. Note how the geodesic is shape-preserving. On the other hand, the geodesic in the Euclidean space is illustrated in panel **b** by means of calculating, for $t\in [0,1]$, the density function $n_t(\,\cdot \,){:}{=}(1-t)n_0(\,\cdot \,)+tn_1(\,\cdot \,)$ (i.e., the segment joining $n_0(\,\cdot \,)$ and $n_1(\,\cdot \,)$), which is clearly not shape-preserving

In Sect. 2.1 we have recalled how Wasserstein distances have become popular due to some intuitive properties they guarantee. In particular, geodesics defined by means of Wasserstein distances are shape-preserving, as Fig. 1 illustrates.

Geodesics are shape-preserving due to the fact that, in some sense, the Wasserstein distance accounts for the form of the underlying distribution. For instance, if one considers three continuous uniform distributions $\mathbb {U}_1$, $\mathbb {U}_2$ and $\mathbb {U}_3$ on [0, 1], [1, 2] and [100, 101], respectively, then by (2) it results that $W_2(\mathbb {U}_1,\mathbb {U}_2)=1$ while $W_2(\mathbb {U}_1,\mathbb {U}_3)=10$. Therefore, distributions with “similar” densities are “close”, according to the (square) Wasserstein distance, while this is not the case if densities are “far” from each other. Note that other metrics between probability distributions would not be sensitive to “geometrical” similarities. For instance, we recall that the *total variation distance* (see Villani 2009, Sect. 6) between two (absolutely continuous) probability measures $\mathbb {P}$ and $\mathbb {Q}$ with densities $f_{\mathbb {P}}(\,\cdot \,)$ and $f_{\mathbb {Q}}(\,\cdot \,)$, respectively, denoted as $TV(\mathbb {P},\mathbb {Q})$, can be computed as $\frac{1}{2}\int _{-\infty }^\infty |f_\mathbb {P}(x)-f_\mathbb {Q}(x)|\,dx$ . Additionally, the *(square) Hellinger distance* (see Le Cam 1986, Sect. 4) between $\mathbb {P}$ and $\mathbb {Q}$, denoted as $H_2(\mathbb {P},\mathbb {Q})$, can be calculated as $\int _{-\infty }^\infty (\sqrt{f_\mathbb {P}(x)}-\sqrt{f_\mathbb {Q}(x)})^2\,dx$. It then results that $TV(\mathbb {U}_1,\mathbb {U}_2)=TV(\mathbb {U}_1,\mathbb {U}_3)=1$ and, similarly, that $H_2(\mathbb {U}_1,\mathbb {U}_2)=H_2(\mathbb {U}_1,\mathbb {U}_3)=1$. The intuitive fact that Wasserstein distances take into account the shape of the probability distributions makes them attractive from the geometrical perspective as well.

As far as exponential distributions are concerned, which are the ones used in this article, we observe that given two exponential distributions with parameters $\lambda$ and $\mu$ (assume $\lambda <\mu$), their total variation distance equals $\left( \frac{\mu }{\lambda } \right) ^{-\frac{\mu }{\mu -\lambda }} - \left( \frac{\mu }{\lambda } \right) ^{-\frac{\lambda }{\mu -\lambda }}$. Further, their (square) Hellinger distance equals $\frac{(\sqrt{\mu }-\sqrt{\lambda })^2}{\lambda +\mu }$. Thus, the expressions just provided would make the problem of finding the optimal hazard rate with respect to the total variation and (square) Hellinger distances more complicated. We also recall that the *Kullback-Leibler divergence* (Kullback and Leibler 1951) between two (absolutely continuous) probability distributions $\mathbb {P}$ and $\mathbb {Q}$ with densities $f_{\mathbb {P}}(\,\cdot \,)$ and $f_{\mathbb {Q}}(\,\cdot \,)$, respectively, is defined as $KL(\mathbb {P},\mathbb {Q})=\int _{-\infty }^{\infty }f_{\mathbb {P}}(x)\ln \left( \frac{f_{\mathbb {P}}(x)}{f_{\mathbb {Q}}(x)}\right) \,dx$. Thus, the Kullback-Leibler divergence is not symmetric. Given $\mathbb {P}$ and $\mathbb {Q}$ exponentially distributed as before (i.e., with rate parameters equal to $\lambda$ and $\mu$, respectively), we obtain that $KL(\mathbb {P},\mathbb {Q})=-\log \frac{\mu }{\lambda }-1+\frac{\mu }{\lambda }$. If in (6) $W^2_2(\mathbb {Q}_i,\mathbb {Q}^*)$ were to be substituted with $KL(\mathbb {Q}_i,\mathbb {Q}^*)$ for every *i*, then the optimal hazard rate would still coincide with the weighted harmonic average (7). On the other hand, if $W^2_2(\mathbb {Q}_i,\mathbb {Q}^*)$ were to be substituted with $KL(\mathbb {Q}^*,\mathbb {Q}_i)$, then the optimal hazard rate would coincide with the weighted arithmetic average of the $\lambda _i$’s. As the $\lambda _i$’s can be interpreted as rates, then the natural way of averaging them is that of considering their (weighted) harmonic mean rather than their (weighted) arithmetic one.

The examples and considerations provided above illustrate how Wasserstein distances are very suitable tools to measure, in an intuitive manner, distances between probability distributions. This makes them suitable tools to be employed for the purpose of computing distances between default probability distributions as proposed in this article, as opposed to some other well-known metrics.

## Examples

In this section we apply the methodology described in this article to two different datasets. We compare the results to those obtained by applying the intersection methodology when the proxy CDS par spread for a given bucket is defined as the arithmetic average of the CDS spreads therein allocated. For the sake of clarity and brevity, in this section we will often refer to the methodology outlined in this article as the *Wasserstein methodology*, while we will refer to the intuitive procedure of arithmetically averaging CDS spreads as the *average methodology*. In both examples considered here we take into account the time period spanning from September 2019 to September 2021. As far as the tenor of the CDS contracts is concerned, we focus our analysis on the 5Y tenor, which is in general the most liquid.

**Fig. 2:**
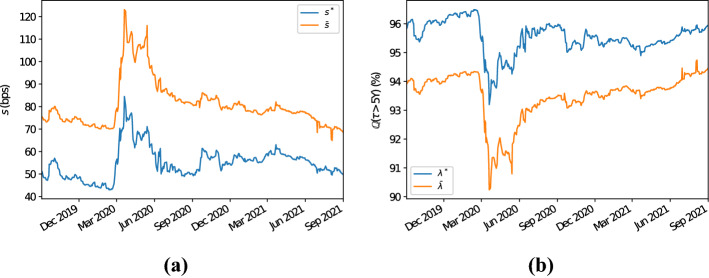
5Y CDS proxy par spreads and survival probabilities for the bucket with region “North America”, sector “financials”, rating “A” and seniority “senior unsecured debt”; see panels **a** and **b**, respectively. The proxy CDS par spreads depicted in panel **a** labelled with $s^*$ ($\bar{s}$) have been computed using the Wasserstein (average) methodology. Similarly, the 5Y proxy survival probabilities of panel **b** labelled with $\lambda ^*$ ($\bar{\lambda }$) are based on the hazard rate calculated with the Wasserstein (average) methodology

As a first example we take into account the bucket where the region is “North America”, the sector “financials”, the rating “A”, and the seniority “senior unsecured debt”. In this bucket, for the time period and the tenor considered, the number of entities oscillates between a minimum of 55 and a maximum of 62. Figure 2a displays the 5Y proxy CDS spreads computed using the Wasserstein methodology and those computed using the average one. It is clear that calculating proxy CDS spreads by means of Wasserstein barycentres produces results that are consistently below their average counterparts. In particular, we observe differences of at least 15–20bps and, during one of the peaks of the Covid-19 pandemic (i.e., in the neighbourhood of the second quarter of 2020), the magnitude of the discrepancies observed reaches values higher than 40bps. The differences observed here are therefore reflected in the associated implied survival probabilities. In fact, as Fig. 2b shows, arithmetically averaging the CDS spreads in the bucket largely underestimates (overestimates) the proxy survival (default) probabilities. In particular, Fig. 2b highlights how, given a 5Y horizon, survival probabilities implied from the averaged CDS quotes differ by at least 1.5–2% from those implied using the Wasserstein methodology, with peaks between 3 and 3.5% during the Covid-19 period mentioned above.

**Fig. 3:**
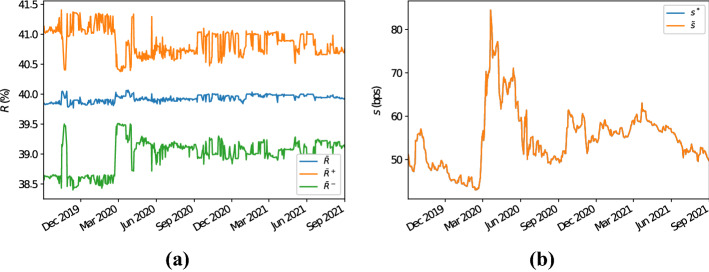
Panel **a** illustrates the 5Y CDS average recovery rates for the bucket with region “North America”, sector “financials”, rating “A” and seniority “senior unsecured debt”. In particular, $\bar{R}$ denotes the arithmetic average of the recovery rates of the CDSs in the chosen bucket, while $\bar{R}^+$ ($\bar{R}^-$) denotes the arithmetic average of the recovery rates of the CDSs in the chosen bucket plus (minus) their standard deviation. Panel **b** displays the 5Y proxy survival probabilities for this bucket calculated with the Wasserstein methodology. The label $s^*$ ($\tilde{s}$) indicates that the proxy recovery rate has been calculated as the arithmetic (harmonic) average of the recovery rates of the CDSs in the bucket considered

The reasons why simply averaging CDS spreads overestimates their proxy counterparts (and therefore, overestimates default probabilities) is due to the fact that, often, recovery rates for CDSs are set equal (or close) to some standard values (see Sect.  3 and Das and Hanouna (2008)). Therefore, CDSs with similar characteristics in terms of sector, region, rating, etc. often share similar recovery rates. In particular, frequently recovery rates are set equal to (or neighbouring) 40%. This is illustrated by Fig. 3a, which shows that the average recovery rate for this bucket often lies between 39.5 and 40.5%, and that it has a small standard deviation. In Sect. 3 we have observed that, if the recovery rates in a given bucket are all equal, then by inverting (5) the resulting proxy CDS par spread equals the harmonic mean of the CDS spreads in the bucket (if all recovery rates are equal and the proxy recovery rate equals their average). Due to the small standard deviation of the recovery rates observed, it results that all the recovery rates for the CDSs in the bucket are roughly equal to each other. Therefore, by inverting (5), the proxy CDS spread for this bucket is approximately equal to the harmonic average of the observed CDS spreads, which is smaller than its arithmetic counterpart. Note that, given the dataset considered, if we compute the proxy recovery rate as the harmonic mean of the observed recovery rates, then results do not change significantly as recovery rates are stable through time; see Fig. 3b. This shows that the methodology is robust with respect to the definition of the proxy recovery rate.

**Fig. 4:**
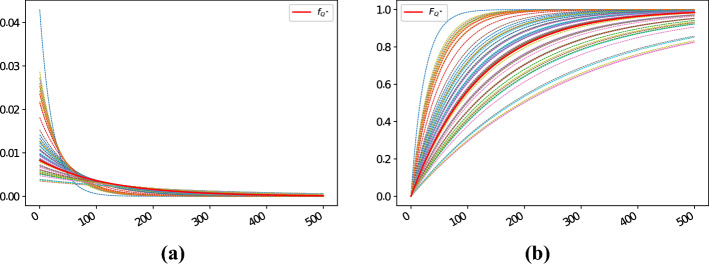
Density and distribution functions implied from the 5Y CDSs with region “North America”, sector “financials”, rating “A” and seniority “senior unsecured debt”, as well as their proxy counterparts (thick red lines) computed using the Wasserstein methodology as of 1-Sep-2021; see panels **a** and **b**, respectively

For completeness, we also illustrate how the density and distribution functions for the default times as of 1-Sep-2021 look like in this specific case, and compare them with their proxy counterparts computed using the Wasserstein methodology; see Fig. 4a and b, respectively.

**Fig. 5:**
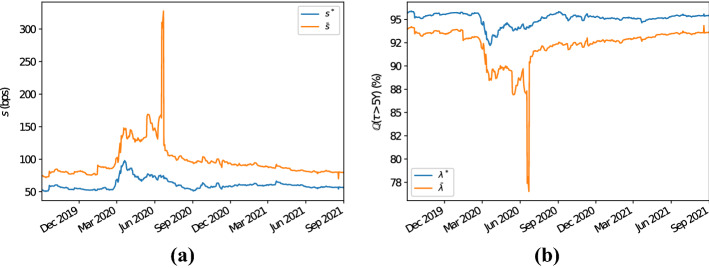
5Y CDS proxy par spreads and survival probabilities for the bucket with region “Europe”, sector “industrials”, rating “BBB” and seniority “senior unsecured debt”; see panels **a** and **b**, respectively. The proxy CDS par spreads depicted in panel **a** labelled with $s^*$ ($\bar{s}$) have been computed using the Wasserstein (average) methodology. Similarly, the 5Y proxy survival probabilities of panel **b** labelled with $\lambda ^*$ ($\bar{\lambda }$) are based on the hazard rate calculated with the Wasserstein (average) methodology

We now consider a second example where the selected CDS bucket is identified by the “Europe” region, “industrials” sector, “BBB” rating and “senior unsecured debt” seniority. The number of constituents for this bucket varies, throughout the period taken into account, between a minimum of 27 and a maximum of 38. We have chosen this bucket as, during the time period considered for the analysis, the dataset shows some outliers. Therefore, this allows to test the robustness of the proposed methodology in these circumstances compared to the standard intersection methodology. As Fig. 5a illustrates, the proxy CDS quotes computed by means of Wasserstein barycentres are, as in the previous example, consistently below their counterparts computed using arithmetic averages. Differences are, for the majority of the dates considered, of the order of magnitude between 20 and 25bps. This is clearly reflected by the related implied survival probabilities, where those implied from arithmetically averaged CDS spreads are underestimated; see Fig. 5b. We observe how, between June and September 2020, the dataset considered displays a peak in the proxy spread (and survival probabilities) in the case proxy CDS spreads are calculated using the average methodology due to some outliers (some other minor peaks are also present in the time frame we consider in this example, but with smaller magnitude). We clearly see, as Fig. 5a and b illustrate, that computing proxy CDS spreads by means of Wasserstein barycentres, results are much more stable. The peak observed in the case the average methodology is used results in a difference in the proxy CDS spread (5Y survival probability) of the order of more than 250bps (15%) compared to the Wasserstein methodology case.

**Fig. 6:**
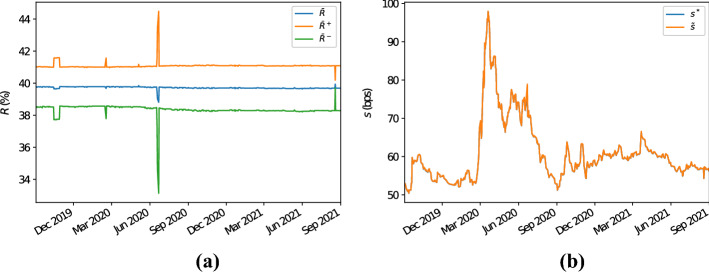
Panel **a** illustrates the 5Y CDS average recovery rates for the bucket with region “Europe”, sector “industrials”, rating “BBB” and seniority “senior unsecured debt”. In particular, $\bar{R}$ denotes the arithmetic average of the recovery rates of the CDSs in the chosen bucket, while $\bar{R}^+$ ($\bar{R}^-$) denotes the arithmetic average of the recovery rates of the CDSs in the chosen bucket plus (minus) their standard deviation. Panel **b** displays the 5Y proxy survival probabilities for this bucket calculated with the Wasserstein methodology. The label $s^*$ ($\tilde{s}$) indicates that the proxy recovery rate has been calculated as the arithmetic (harmonic) average of the recovery rates of the CDSs in the bucket considered

Also in this case we observe that, as expected, for the CDSs in the chosen bucket recovery rates do not vary much, with a few exceptions; see Fig. 6a. Therefore, also in these circumstances the same rationale as outlined in the former example can be followed to explain why proxy CDS spreads calculated with the average methodology (and corresponding default probabilities) overestimate their counterparties computed following the Wasserstein methodology. We also show, see Fig. 6b, that the Wasserstein methodology is robust with respect to the averaging type used to compute the proxy recovery rate (i.e., arithmetic or harmonic).

**Fig. 7:**
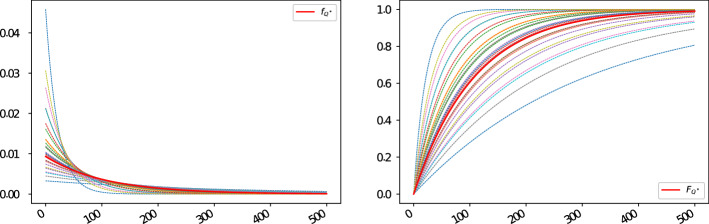
Density and distribution functions implied from the 5Y CDSs with region “Europe”, sector “industrials”, rating “BBB” and seniority “senior unsecured debt”, as well as their proxy counterparts computed using the Wasserstein methodology as of 1-Sep-2021 (thick red lines); see panels **a** and **b**, respectively

Again, for completeness we also illustrate how the density and distribution functions for the default times as of 1-Sep-2021 look like in this second example as well, and how they compare with respect to their proxy counterparts computed by means of the Wasserstein methodology; see Fig. 7a and b, respectively.

We have provided a comparison between a common way to compute proxy CDS spreads and related credit curves (i.e., intersection approach with average methodology) and those computed following the approach proposed based on Wasserstein distances. We have shown how, by considering real market scenarios, the differences in default probabilities produced using the two methodologies are non-marginal. In particular, given the examples taken into account, we have shown how proxying CDS spreads by means of the average methodology consistently overestimates the resulting implied survival probabilities. This means that simply arithmetically averaging CDS spreads can produce unjustifiably conservative survival probabilities. This is due to the fact that, despite simple and intuitive, this approach lacks of adequate theoretical foundations. As proxy survival probabilities are often used by financial institution to compute Credit Valuation Adjustment (CVA), using the intersection approach with the average methodology given the datasets considered here would result in excessively conservative CVA charges compared to those calculated using proxy survival probabilities computed using the Wasserstein methodology. Thus, under market circumstances for which the examples taken into account here can be considered representative, the Wasserstein methodology would result in more aggressive CVA charges than those computed when the average methodology is used.

## Conclusion

In this article we have investigated an alternative way of estimating proxy CDS curves starting from the intersection methodology. In particular, instead of simply averaging the different CDS quotes belonging to the given buckets, we have constructed, under some simple assumptions, a proxy CDS curve by means of an optimal transportation problem. That is, after having specified a given functional form for the CDS proxy curve to be estimated, we have provided simple formulae that allow to calculate the proxy hazard rates (and, thus, proxy par spreads) by simply minimizing the Wasserstein square distances between the proxy curve and the other curves belonging to the chosen bucket. The approach we have adopted is simple and relies on basic assumptions concerning CDSs that are often employed in practice. It also provides a simple interpretation of the proxy CDS default distribution as the actual Wasserstein barycenter of the observed default probability distributions.

## Footnotes

1 In principle, more granular bucketing can take place, but in this article we do not deal with the choice of the bucketing methodology, and we consider it as given.

2 Therefore, from here onwards we will often not refer explicitly to the choice of the buckets as, from the implementation angle, this does not matter. We will thus often use more general expressions such as, for instance, “bucketing methodology”, for brevity.

3 Note that more general conditions are sufficient for the existence and uniqueness of a Wasserstein barycenter; see Agueh and Carlier (2011). However, for our purpose of constructing proxy CDS curves we will be working with absolutely continuous probability measures only.

4 For simplicity, from here onward we will refer to (3) simply as Wasserstein barycenter.

5 Note that we have excluded the case where $\lambda (t)=0$ as this corresponds to the (instantaneous) default-free case.

6 The recovery rate *R* is assumed exogenously given. Further details can be found in Das and Hanouna (2008).

7 For a derivation of the credit triangle relationship see, for instance, White (2014).

8 Also in this case, the derivation of the credit triangle relationship can be found in White (2014).

9 Note that, in the case of uniform weights, the weights would be redundant. For completeness, however, we explicitly consider the weights in (6). This is because, in practice, if a bucket only contains a handful of names, then the modeler might want to add to the bucket some other CDSs (for instance with the same sector and rating but belonging to a different region) and assign them a small weight to have more stable results. However, as mentioned in the beginning of the section, in this article a bucketing methodology is assumed as given. We provide the formulation (6) (as well as that of weighted Wasserstein barycenter (3)) as they are slightly more general than the uniform case, allowing thus for more flexibility.

10 Note that, in principle, also other parametrizations of the hazard rate functions are possible, such as cubic spline.

11 In principle, recovery rates can vary. However, in many cases fixed values for recovery rates are used; see Das and Hanouna (2008).
